# Observation of Hyperpositive Non-Linear Effect in Asymmetric Organozinc Alkylation in Presence of *N*-Pyrrolidinyl Norephedrine

**DOI:** 10.3390/molecules27123780

**Published:** 2022-06-11

**Authors:** Thibault Thierry, Yannick Geiger, Stéphane Bellemin-Laponnaz

**Affiliations:** 1 Institut de Physique et Chimie des Matériaux de Strasbourg, Université de Strasbourg-CNRS UMR7504, 23 rue du Loess, BP 43, CEDEX 2, 67034 Strasbourg, France; thibault.thierry@ipcms.unistra.fr (T.T.); y.geiger@rug.nl (Y.G.); 2 Stratingh Institute for Chemistry, University of Groningen, Nijenborgh 4, 9747 AG Groningen, The Netherlands

**Keywords:** asymmetric catalysis, nonlinear effect, chiral amplification

## Abstract

Phenomena related to asymmetric amplification are considered to be key to understanding the emergence of homochirality in life. In asymmetric catalysis, theoretical and experimental models have been studied to understand such chiral amplification, in particular based on non-linear effects. Three decades after the theoretical demonstration that a chiral catalyst, when not enantiopure, could be more enantioselective than its enantiopure counterpart, we show here a new experimental example of nonlinear hyperpositive effect. We report here our investigations in the enantioselective zinc-catalyzed alkylation of benzaldehyde with *N*-pyrrolidinyl norephedrine as partially resolved chiral ligand, which shows a significant hyperpositive non-linear effect. A study of the underlying mechanism was conducted, which allows us to confirm a mechanism that implies a monomeric and a dimeric complex both catalyzing the reaction at a steady state and giving different enantioselectivities.

## 1. Introduction

**Figure 1:**
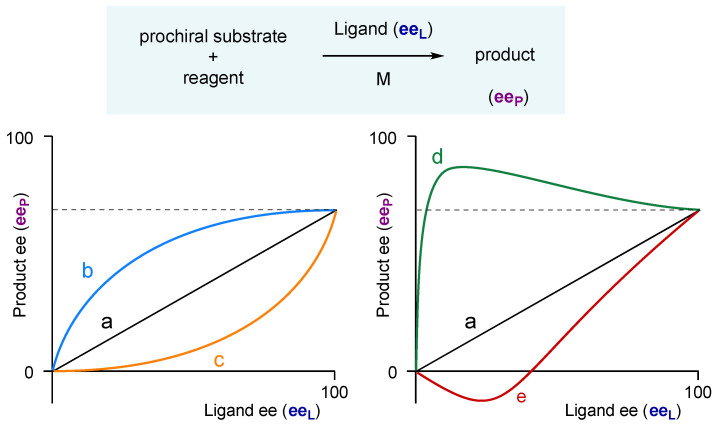
Product ee vs. ligand ee graphs of catalytic asymmetric reactions showing examples for (**a**) no NLE, (**b**) a positive NLE ((+)-NLE), (**c**) a negative NLE ((−)-NLE), (**d**) a hyperpositive NLE and (**e**) an enantiodivergent NLE.

Asymmetric synthesis and catalysis are of paramount importance for obtaining enantiopure molecules, especially in the development of chiral drugs that are almost exclusively applied in the form of single enantiomers [1]. In asymmetric catalysis, a chiral auxiliary (most often a chiral ligand attached to a metal) is used ideally in its enantiomerically pure form to maximize the enantiomeric excess of the desired product (ee_P_). When the chiral auxiliary is not enantiopure, in most cases a linearity is observed between the optical purity of the product (ee_P_) and the reagent (ee_L_), as shown in Figure 1a. However, deviations from linearity between ee_P_ and ee_L_ can occur and are referred to as non-linear effects (NLEs) [2,3]. In the case of a positive non-linear effect (often called (+)-NLE), the ee_P_ can be much higher than the ee_L_, resulting in asymmetric amplification (Figure 1b). The opposite is also possible, called negative non-linear effect ((−)-NLE) (Figure 1c).

Recently, we observed that a chiral catalyst, when not enantiopure, can be more enantioselective than its enantiopure counterpart (Figure 1d) [4]. Such an unusual, “hyperpositive” NLE was first suggested by Kagan in the mid-90s [2,5,6]. This was observed in the zinc-catalyzed alkylation of aldehyde in the presence of N-benzyl ephedrine, and we rationalized the hyperpositive NLE by introducing a monomer-dimer competition model, where both monomers and dimers are catalytically active [4,7,8,9]. Such a model also allows us to describe enantiodivergent NLEs, that is, when a catalyst can generate one enantiomer or its opposite by simply varying the ligand ee (see Figure 1e), which was observed with N-methyl ephedrine as ligand [10].

Since chiral ligands based on ephedrine or norephedrine have been and still are widely used in asymmetric catalysis, it appeared important to study other derivatives of this family in the context of non-linear effect. Among all the derivatives, *N*-pyrrolidinyl norephedrine (NPNE) attracted our attention [11,12] because it has not been probed yet for NLEs in a systematic way, although it is one of the most enantioselective ligands in ephedrine-based catalytic organozinc additions.

Herein, we wish to report our investigations in the enantioselective zinc-catalyzed alkylation of benzaldehyde with NPNE as a partially resolved chiral ligand. A hyperpositive non-linear effect was observed with diethylzinc or dimethylzinc as the reagent, which was found to be more pronounced at low temperatures. The results were rationalized by studying the effect of temperature and concentration and on the basis of our previous studies with N-benzyl ephedrine. Overall, this system concords with the previously proposed monomer-dimer competition model, where a monomeric species is in equilibrium with a dimeric species, both being active and competing to generate the product, albeit with different enantioselectivity.

## 2. Results

### 2.1. Non-Linear Studies

**Figure 2:**
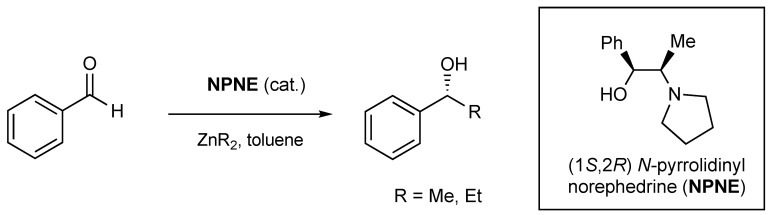
Catalytic enantioselective addition of dialkylzincs to benzaldehyde, catalyzed by *N*-pyrrolidinyl norephedrine.

**Figure 3:**
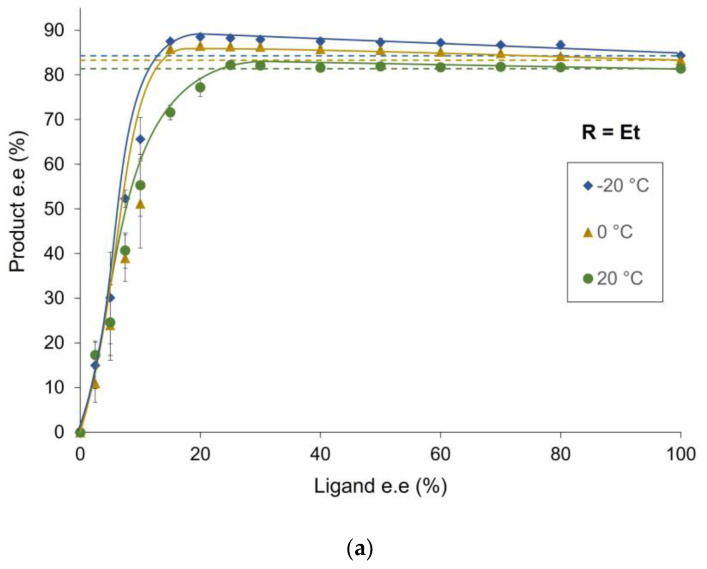

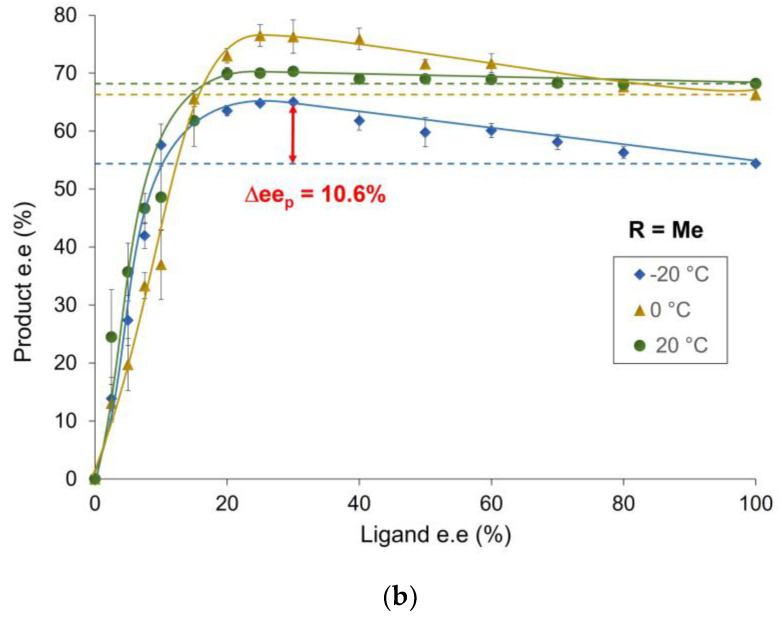
Optical purity of the product as a function of the enantiomeric excess of NPNE ligand (20 mol%) at different temperatures. (**a**) with ZnEt_2_ as reagent; (**b**) with ZnMe_2_ as reagent. The reaction conditions and experimental procedure are described in the Supplementary Methods. Each point is the mean of three different experiments. The vertical error bars depict standard deviations. The dashed line represents the product e.e. of the enantiopure compound; the full line is a free-hand drawing, which serves as a guideline.

The zinc-catalyzed alkylation of benzaldehyde was investigated with partially resolved chiral *N*-pyrrolidinyl norephedrine ligand (NPNE; Figure 2). Figure 3 displays the results with diethylzinc (a) or dimethylzinc (b) as the reagent. The catalyst loading was fixed at 20 mol%, and toluene was used as solvent. The reactions were carried out at 20 °C, 0 °C and −20 °C.

In case (a), at room temperature, the product was isolated in 81.4% ee when the enantiopure ligand was used (green curve). A strong positive non-linear effect occurred with essentially no change of the product enantiopurity up to ee_L_ of 25%. The lowering of the reaction temperature induced a slight increase in the optical purity of the product and the appearance of a hyperpositive effect that is more significant at −20 °C than 0 °C (Δee of 4.2% and 3.2%, respectively). In contrast, we observed an inversed tendency in case (b): lowering the temperature decreased also the enantioselectivity with enantiopure ligand, with a significant drop between 0 and −20 °C [from 66.3% to 54.4%], and increased the hyperpositive NLE up to a Δee of 10.6% at −20 °C. 

**Figure 4:**
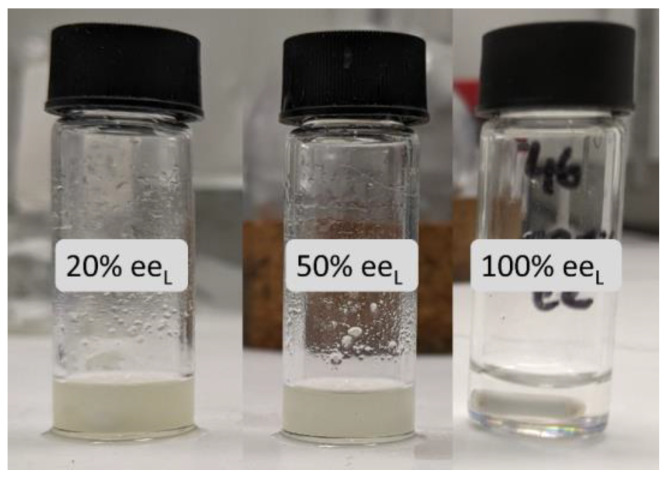
Reaction medium (ligand, substrate, diethylzinc, toluene) as function of the optical purity of the NPNE ligand. From left to right: 20% ee_L,_ 50% ee_L_ and 100% ee_L_.

During the catalytic investigations, we detected that, when the partially resolved ligand was used, the formation of a precipitate upon addition of the dialkylzinc reagent. This was in contrast to the use of the enantiopure ligand, which gave a clear solution. Figure 4 displays three selected catalytic samples at 20%, 50% and 100% ee_L_ (from left to right) that clearly illustrates the differences. Additional experiments were carried out to identify the nature of the precipitate. Analyses of the hydrolyzed precipitate obtained from 20% ee NPNE ligand sample revealed the free ligand in its racemic form, as deduced from the measurement of the optical rotatory power.

### 2.2. Impact of Catalyst Loading

**Figure 5:**
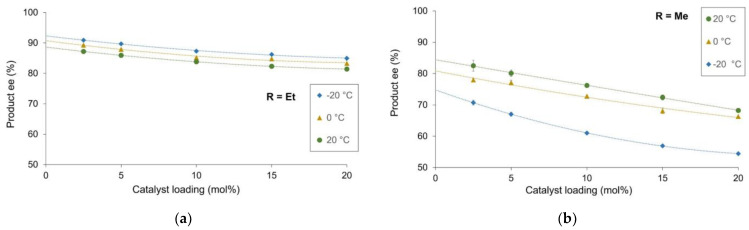
Optical purity of the product as a function of the catalyst loading of NPNE ligand (enantiopure 1*S*-2*R* form) at different temperatures. (**a**) with ZnEt_2_ as reagent; (**b**) with ZnMe_2_ as reagent. The reaction conditions and experimental procedure are described in the Supplementary Methods. Each point is the mean of three different experiments. The vertical error bars depict standard deviations. The dotted trendlines are second-order polynomial fits, which serve as guidelines.

The enantioselective reaction was evaluated by varying the catalyst loading of the enantiopure NPNE ligand with diethylzinc (Figure 5a) or dimethylzinc (Figure 5b) as reagent. In both cases, the results showed that a decrease in catalyst loading resulted in an increase in the enantiomeric excess of the product. 

All curves show the same trend, the major difference between the two reagents lies in the temperature effect as already observed in the previous NLE studies.

### 2.3. Impact of Temperature

**Figure 6:**
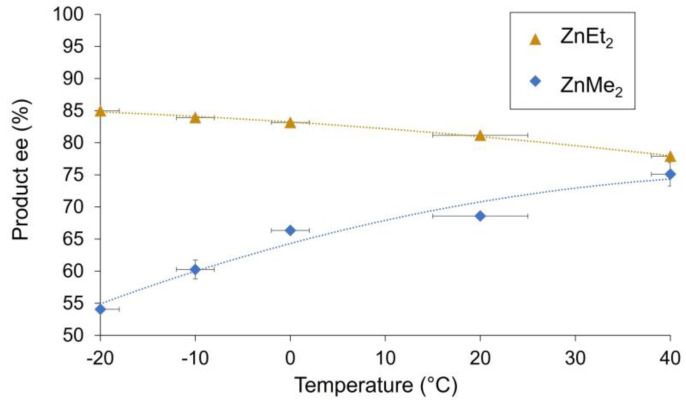
Optical purity of the product as a function of the temperature. Orange triangles correspond to the reaction with ZnEt_2_ as reagent; blue squares correspond to the reaction with ZnMe_2_ as reagent. The reaction conditions and experimental procedure are described in the Supplementary Methods. Each point is the mean of three different experiments. The horizontal error bars depict temperature variation during the reaction. The vertical error bars depict standard deviations. The dotted trendlines are second-order polynomial fits, which serve as guidelines.

The impact of the reaction temperature on the product enantioselectivity was investigated. Figure 6 displays the results ranging from −20 °C to 40 °C with diethylzinc (yellow triangle) or with dimethylzinc (blue square) as the reagent. With diethylzinc, the product ee went down from 84.4% at −20 °C to 77.8% at 40 °C. In contrast, the product ee increased from 54.4% at −20 °C to 72.4% at 40 °C with dimethylzinc. 

## 3. Discussion

The norephedrine skeleton has been used extensively for the development of efficient chiral auxiliaries in asymmetric synthesis and asymmetric catalysis, mostly with zinc as the active metal [13,14]. Enantioselectivities greater than 90% ee were often reported in the alkylation (or alkynylation) of carbonyl or imine compounds [15,16,17]. Additionally, Efavirenz, an industrially produced drug for the treatment of Human Immunodeficiency Virus (HIV), includes an enantioselective transformation mediated by *N*-pyrrolidinyl norephedrine (NPNE) [18]. We therefore decided to focus NLE studies with NPNE ligand to better understand the nature of the catalytically active species.

Investigations of the product ee (ee_P_) as function of ligand ee (ee_L_) with NPNE revealed a hyperpositive NLE in the zinc-catalyzed alkylation reaction (Figure 3). Such behavior is similar to what was observed previously with the *N*-benzyl ephedrine ligand [4]. Interestingly, using ZnMe_2_ as reagent the product enantioselectivities are higher than with the NBE ligand. The hyperpositive NLE effects (i.e., the difference between the highest ee_P_ obtained and the ee_P_ with enantiopure ligand) with NPNE ligand are relatively small at room temperature and more pronounced at lower temperature (0 and −20 °C).

Similar to what was observed with the NBE system, a zinc aggregate precipitated when the ligand was not enantiopure, as shown in Figure 4 [19]. A measurement of the rotatory power of the ligand contained in the aggregate revealed that the ligand was in its racemic form. Therefore, the precipitate is an overall racemic mixture of NPNE complexes, as previously observed with NBE as ligand, most likely as a heterochiral dimer or co-precipitated RRS·RSS trimer adduct (trimeric adducts were observed in solution with the parent ligand *N*-methyl ephedrine) [8]. Such precipitation of racemic ligand causes a strong positive non-linear effect to emerge, as it enantioenriches the zinc species left in solution (i.e., Kagan’s *reservoir effect*) [3]. Trapping the ligand in a racemic form implies that, when decreasing the enantiomeric excess of the ligand, we generate more racemic inactive aggregates and thus less catalytically active species. Figure 5 displays the evolution of product ee as a function of the catalyst loading of the enantiopure NPNE. In all cases, a decrease of the catalyst loading led to an increase of the product ee. 

**Figure 7:**
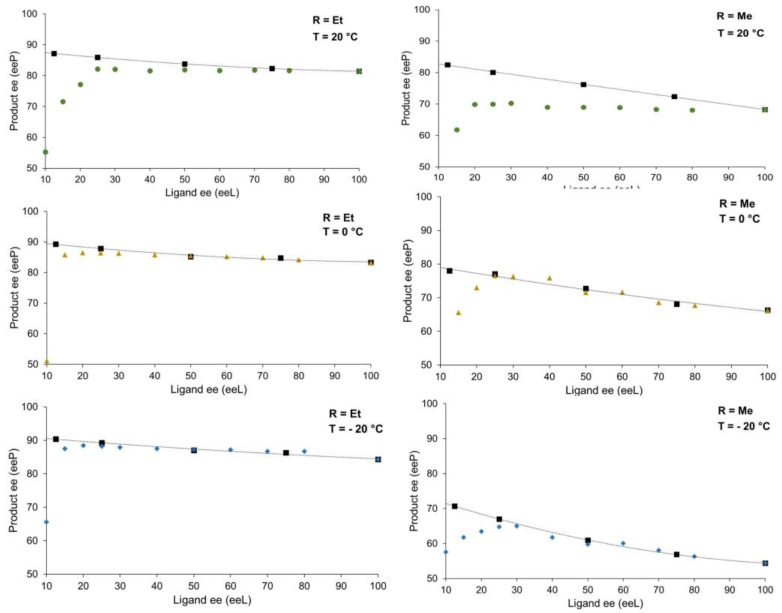
Superimposition of the non-linear effect studies from Figure 2 (colored dots, triangles, and tilted quads) and the ee_P_ vs catalyst loading data from Figure 4 (black quads) that was converted to ee_P_ vs. simulated ligand ee, assuming a complete trapping of the racemic ligand.

The catalytic loading effect with the enantiopure ligand and the non-linear effects were correlated. Considering that the reservoir effect on the racemic species is optimal in these systems, we could correlate the data points from the catalyst loading effect with a virtual enantiomeric excess if a scalemic ligand was used. The plots were superimposed with the NLE curves and are displayed in Figure 7 below [4]. At −20 °C and 0 °C, good agreement was observed but not at room temperature.

**Figure 8:**
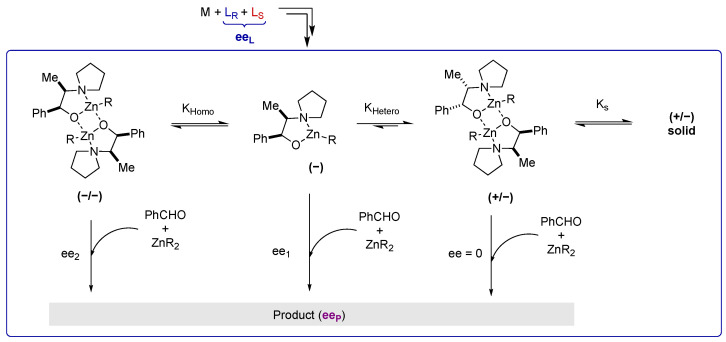
Proposed enantiodivergent model with (−)-NPNE as major enantiomer.

Altogether, these results indicate a monomer-dimer mechanism as proposed in Figure 8 [20]. Two different enantiodivergent pathways are present in the system: a monomeric (−)-NPNE-ZnR-catalyzed mechanism and a dimeric (−)-NPNE-ZnR-catalyzed mechanism that operate at a steady state; the minor (+)-enantiomer is trapped within a racemic dimer and a racemic precipitate. Since the overall ee (and yield) of the product is the combination of the two catalysts, if (i) the monomeric catalyst is more enantioselective than the dimeric catalyst (i.e., ee_1_ > ee_2_), (ii) the two chiral catalysts are in dynamic equilibrium and (iii) the minor enantiomer is trapped in the form of an inactive species such as a solid heterochiral dimer, then a hyperpositive non-linear effect is possible. Indeed, decreasing ee_L_ leads to an increase of ee_P_ because the equilibrium should be shifted to the monomeric catalyst. Decreasing the catalyst loading should have the same effect. 

However, this hyperpositive non-linear effect will only be possible if the reservoir effect is highly efficient, trapping the minor enantiomer. This is the case at low temperatures (0 °C and −20 °C). On the other hand, at room temperature, we observe essentially a “classical” positive non-linear effect, although the study of the catalysis revealed an increase of ee_P_ as function of the catalyst loading (Figure 4). This suggests that, at room temperature, a portion of the heterochiral dimer is soluble and lowers the overall ee_P_ by allowing for the formation of the minor enantiomer catalyst, or by being catalytically active itself and generating racemic product. 

In asymmetric catalysis, the enantioselectivity is determined by the relative rates of the enantio-differentiating step (i.e., ΔΔG*), which is temperature dependent. As a result, the enantioselectivity is greater at lower temperature. Here, we also investigated the effect of the temperature on the overall product ee of the reaction (Figure 5). With diethylzinc as the reagent, ee_P_ goes from 84.2% at −20 °C to 77.8% at 40 °C, a variation that is very little given the temperature range. With dimethylzinc, the enantiomeric excess of the product was significantly increased (from 54.4% to 75.1%), which is an unusual inverse temperature dependence. Altogether, this is consistent with an equilibrium between a monomeric species and a dimeric species, which is shifted towards the more enantioselective monomer at higher temperature.

## 4. Materials and Methods

Synthetic procedures as well as additional data for experimental catalytic runs can be found in the Supplementary Information Section, including detailed experimental data (reactant quantities, reaction conditions, raw and treated results for all catalytic runs).

## 5. Conclusions

In asymmetric synthesis or catalysis, the NPNE ligand is one of the most used in the family of ephedrine-based ligands usually generating products with high enantiocontrol. The study of the ee of the product versus the ee with this ligand in the asymmetric organozinc alkylation demonstrated that the chiral catalyst, when not enantiopure, is more enantioselective than its enantiopure counterpart. Mechanistic studies show that the system follows the same pattern as that with N-benzyl ephedrine or N-methyl ephedrine ligands: with the NPNE ligand, we noticed the presence of an insoluble racemic aggregate that allowed us to trap the minor enantiomer catalyst. The remaining enantio-enriched active species consist in a two-component system, where a monomeric catalyst is in equilibrium with a dimeric catalyst. The equilibrium between the catalytic species is dependent on the total concentration of species in solution, which depends on the enantiomeric excess of the ligand used in the reaction. Evidence for this model has been found by varying the catalyst loading or the reaction temperature. A correlation between the catalytic loading effect with the enantiopure ligand and the non-linear effects suggested that part of the racemic aggregate could catalyze the reaction and reduce the magnitude of the observed hyperpositive non-linear effect at higher temperature.

## Data Availability

Data are contained within the article and Supplementary Materials.

## Supplementary Materials

The following supporting information can be downloaded at: https://www.mdpi.com/article/10.3390/molecules27123780/s1, Table S1: Detailed ligand, benzaldehyde, ZnEt2 and solvent quantities for NLEs study; Table S2: Detailed ligand, benzaldehyde, ZnMe2 and solvent quantities for NLEs study; Table S3: Detailed ligand, benzaldehyde, ZnEt2 and solvent quantities for the catalyst loading screening; Table S4: Detailed ligand, benzaldehyde, ZnMe2 and solvent quantities for the catalyst loading screening; Table S5: Detailed ligand, benzaldehyde, ZnEt2 and solvent quantities for the temperature screening; Table S6: Detailed ligand, benzaldehyde, ZnMe2 and solvent quantities for the temperature screening; Table S7: Reported data from NLEs studies using ZnEt2 at different temperatures; Table S8: Reported data from NLEs studies using ZnMe2 at different temperatures; Table S9: Reported data from catalyst loading screening using ZnEt2 at different temperatures; Table S10: Reported data from catalyst loading screening using ZnMe2 at different temperatures; Table S11: Reported superimposition data from ZnEt2 use; Table S12: Reported superimposition data from ZnMe2 use; Table S13: Reported data from the temperature screening.
