# Correction: Thierry et al. Observation of Hyperpositive Non-Linear Effect in Asymmetric Organozinc Alkylation in Presence of *N*-Pyrrolidinyl Norephedrine. *Molecules* 2022, *27*, 3780

**DOI:** 10.3390/molecules29174265

**Published:** 2024-09-09

**Authors:** Thibault Thierry, Yannick Geiger, Stéphane Bellemin-Laponnaz

**Affiliations:** 1 Institut de Physique et Chimie des Matériaux de Strasbourg, Université de Strasbourg-CNRS UMR7504, 23 rue du Loess, BP 43, CEDEX 2, 67034 Strasbourg, France; thibault.thierry@ipcms.unistra.fr (T.T.); y.geiger@rug.nl (Y.G.); 2 Stratingh Institute for Chemistry, University of Groningen, Nijenborgh 4, 9747 AG Groningen, The Netherlands

The authors wish to make the following correction to their paper [1]:

One reference was missing from the manuscript. Reference 14 should be completed according to the following reference: Soai, K.; Yokoyama, S.; Hayasaka, T. Chiral N,N-Dialkylnorephedrines as Catalysts of the Highly Enantioselective Addition of Dialkylzincs to Aliphatic and Aromatic Aldehydes. The Asymmetric Synthesis of Secondary Aliphatic and Aromatic Alcohols of High Optical Purity. *J. Org. Chem.* **1991**, *56*, 4264–4268. https://doi.org/10.1021/jo00013a035. The corresponding citation is added in Section 3.

Reference 13 is a general review of the subject. The added reference is the first example of the use of the ligand N-Pyrrolidinyl-Norephedrine in the asymmetric alkylation of organozinc.

The change does not affect the scientific results. The authors would like to apologize for any inconvenience caused to the readers by this change.
