# Supplementary material for: Observation of Hyperpositive Non-Linear Effect in Asymmetric Organozinc Alkylation in Presence of N-Pyrrolidinyl Norephedrine
Source: Molecules. 2022 Jun 11;27(12):3780. doi: 10.3390/molecules27123780 (PMC9230045; doi:10.3390/molecules27123780)
Supplement: Supplementary file 1 [file molecules-27-03780-s001.zip › molecules-1754582-supplementary.pdf]

Supporting information of

Observation of Hyperpositive Non-Linear Effect in Asymmetric Organozinc Alkylation in Presence of N-Pyrrolidinyl Norephedrine

Thibault Thierry [1], Yannick Geiger [1,2] and Stéphane Bellemin-Laponnaz [1,*]

1 Institut de Physique et Chimie des Matériaux de Strasbourg, Université de Strasbourg-CNRS UMR7504, 23 rue du Loess, BP 43, 67034 Strasbourg Cedex 2, France; thibault.thierry@ipcms.unistra.fr

2 New address : Stratingh Institute for Chemistry, University of Groningen, Nijenborgh 4, 9747 AG, The Netherlands; y.geiger@rug.nl

* Correspondence: bellemin@unistra.fr

Summary

1) Experimental procedures 2

2) Gas Chromatography analysis setting 2

3) General procedure – NPNE-catalysed addition of dialkylzincs to benzaldehyde. 3

4) Non-Linear effects (NLEs) studies 3

5) Catalyst loading screening 4

6) Temperature Study 4

7) Reported Data Tables 5

   a) NLEs data 5

   b) Catalyst loading data 7

   c) Superimposition of ee_P as function of catalyst loading data (from Figure 7 in the paper) 8

   d) Temperature data 10

1) Experimental procedures

All reagents, including (1*S*,2*R*)-*N*-pyrrolidinylnorephedrine (NPNE) and (1*R*,2*S*)-NPNE, were purchased from commercial chemical suppliers (Acros, Alfa Aesar, Sigma-Aldrich and TCI Europe) and used without further purification, except for benzaldehyde, which was distilled and stored under argon prior to use. Enantiomeric excesses were determined on a Varian 3900 gas chromatograph equipped with a CP-8400 autosampler and a Chiraldex G-TA capillary column (25 m long, 250 µm in diameter) with helium as the carrier gas. The reaction temperature was monitored with a HUBER TC45E-F Immersion Chiller.

2) Gas Chromatography analysis setting

Oven temperature: 100°C; Helium pressure: 16 psi; split: 1/20. The separation conditions were determined by injecting racemic versions of the corresponding product. The product's absolute configuration was deducted from earlier reports using the same catalyst.[1]

Note: benzyl alcohol is formed as a side-product in dialkylzinc additions, which is occasionally seen as a trace peak in the GC chromatograms (retention time slightly lower than the 1-phenyl-1-ethanol products).

[1] Geiger, Y., Achard, T., Maisse-François, A. *et al.* Hyperpositive nonlinear effects in asymmetric catalysis. *Nat Catal* **3,** 422–426 (2020). https://doi.org/10.1038/s41929-020-0441-1

3) General procedure—NPNE-catalyzed addition of dialkylzincs to benzaldehyde.

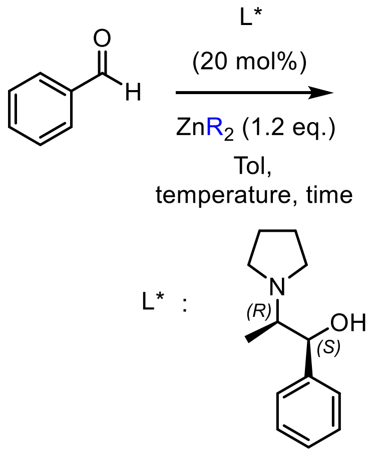

In a N_2-filled glovebox, (1*S*,2*R*)-*N*-pyrrolidinylnorephedrine (20 mol%) and a magnetic stirring bar were placed in an oven-dried vial, which was then closed with a septum-containing screwcap. The vial was put out of the glovebox and a 15% (1M) $ZnEt_2$ solution in toluene (1.2 equiv) was added via syringe; gas evolution occurred. After dry toluene addition*, the mixture was stirred for 10 min, then was set to the desired temperature, and benzaldehyde (1 equiv) was added via syringe. The yellow solution was stirred overnight at the same temperature and turned colorless, and then was quenched carefully with 3M aqueous HCl in an ice-water bath under vigorous stirring. The organic phase was isolated, dried over Na_2SO_4 and analyzed by chiral stationary phase GC.

* A dry toluene addition was realized to obtain a C_{PhCHO}= 0.36M.

<u>Notes</u>: $ZnMe_2$ was used as a 1.2 M solution in toluene (1.2 equiv) with adapted ligand (20 mol%), dry toluene (C_{PhCHO}= 0.36M) and benzaldehyde quantities (1 equiv). Reaction media with $ZnMe_2$ stayed colorless and were left to stir for 3 days.

4) Non-Linear effects (NLEs) studies

For NLEs studies, the ligand e.e. was adjusted by preparing appropriate mixtures of the (1*S*,2*R*) and (1*R*,2*S*)-enantiomers. The general procedure was next applied with adapted ligand (20 mol%), toluene, benzaldehyde and dialkylzinc quantities.

Supplementary Table S1. Detailed ligand, benzaldehyde, $ZnEt_2$ and solvent quantities for NLEs study.

Catload (mol%)	m Ligand (mg)	n Ligand (mmol)	n PhCHO (mmol)	V PhCHO (µL)	nb eq. ZnEt2	n ZnEt2 (mmol)	V ZnEt2 (µL)	C PhCHO (mol/L)	Desired C PhCHO (mol/L)	Toluene add. (µL)
20	12	0.06	0.3	31	1.2	0.36	360	0.83	0.36	473

Supplementary Table S2. Detailed ligand, benzaldehyde, $ZnMe_2$ and solvent quantities for NLEs study.

Catload (mol%)	m Ligand (mg)	n Ligand (mmol)	n PhCHO (mmol)	V PhCHO (µL)	nb eq. ZnMe2	n ZnMe2 (mmol)	V ZnMe2 (µL)	C PhCHO (mol/L)	Desired C PhCHO (mol/L)	Toluene add. (µL)
20	12	0.06	0.3	31	1.2	0.36	300	1.00	0.36	533

5) Catalyst loading screening

The general procedure was applied with the enantiopure (1*S*,2*R*)-NPNE ligand at various catalyst loading, toluene, benzaldehyde and dialkylzinc quantities.

Supplementary Table S3. Detailed ligand, benzaldehyde, $ZnEt_2$ and solvent quantities for the catalyst loading screening.

Catload (mol%)	m Ligand (mg)	n Ligand (mmol)	n PhCHO (mmol)	V PhCHO (µL)	nb eq. ZnEt2	n ZnEt2 (mmol)	V ZnEt2 (µL)	C PhCHO (mol/L)	Desired C PhCHO (mol/L)	Toluene add. (µL)
2.5	3	0.013	0.5	51	1.03	0.51	513	0.98	0.36	876
5	5	0.025	0.5	51	1.05	0.53	525	0.95	0.36	864
10	10	0.050	0.5	51	1.10	0.55	550	0.91	0.36	839
15	15	0.075	0.5	51	1.15	0.58	575	0.87	0.36	814
20	21	0.100	0.5	51	1.20	0.60	600	0.83	0.36	789

Supplementary Table S4. Detailed ligand, benzaldehyde, $ZnMe_2$ and solvent quantities for the catalyst loading screening.

Catload (mol%)	m Ligand (mg)	n Ligand (mmol)	n PhCHO (mmol)	V PhCHO (µL)	nb eq. ZnMe2	n ZnMe2 (mmol)	V ZnMe2 (µL)	C PhCHO (mol/L)	Desired C PhCHO (mol/L)	Toluene add. (µL)
2.5	2	0.01	0.4	41	1.025	0.41	342	1.17	0.36	769
5	4	0.02	0.4	41	1.05	0.42	350	1.14	0.36	761
10	8	0.04	0.4	41	1.1	0.44	367	1.09	0.36	744
15	12	0.06	0.4	41	1.15	0.46	383	1.04	0.36	728
20	16	0.08	0.4	41	1.2	0.48	400	1.00	0.36	711

6) Temperature Study

The general procedure was applied with the enantiopure (1*S*,2*R*)-NPNE ligand at various temperature.

Supplementary Table S5. Detailed ligand, benzaldehyde, $ZnEt_2$ and solvent quantities for the temperature screening.

Catload (mol%)	m Ligand (mg)	n Ligand (mmol)	n PhCHO (mmol)	V PhCHO (µL)	nb eq. ZnEt2	n ZnEt2 (mmol)	V ZnEt2 (µL)	C PhCHO (mol/L)	Desired C PhCHO (mol/L)	Toluene add. (µL)
20	12	0.06	0.3	31	1.2	0.36	360	0.83	0.36	473

Supplementary Table S6. Detailed ligand, benzaldehyde, $ZnMe_2$ and solvent quantities for the temperature screening.

Catload (mol%)	m Ligand (mg)	n Ligand (mmol)	n PhCHO (mmol)	V PhCHO (µL)	nb eq. ZnMe2	n ZnMe2 (mmol)	V ZnMe2 (µL)	C PhCHO (mol/L)	Desired C PhCHO (mol/L)	Toluene add. (µL)
20	12	0.06	0.3	31	1.2	0.36	300	1.00	0.36	533

7) Reported Data Tables

a) NLEs data

Supplementary Table S7. Reported data from NLEs studies using $ZnEt_2$ at different temperatures.

ZnEt2					
20 °C					
At C_{PhCHO} = 0,36 M, rt, 24 h					
Ligand ee (%)	Product ee (%) Run 1	Product ee (%) Run 2	Product ee (%) Run 3	Final product ee (%)	Standard deviation
0	0	0	0	0.0	0.00
2.5	20.87	12.56	18.45	17.3	3.16
5	11.98	25.98	35.9	24.6	8.43
7.5	35.78	46.7	39.76	40.7	3.97
10	53	47.19	65.71	55.3	6.94
15	69.74	71	74.12	71.6	1.67
20	75.03	76.29	80.3	77.2	2.06
25	82.16	82.04	82.34	82.2	0.11
30	81.82	82.39	82.01	82.1	0.21
40	80.66	82.2	82	81.6	0.64
50	81.61	82.74	81.48	81.9	0.53
60	82.07	81.97	81.11	81.7	0.40
70	82.2	81.73	81.46	81.8	0.27
80	82.65	81.33	81.06	81.7	0.65
100	81.78	81.15	81.35	81.4	0.24
0 °C					
At C_{PhCHO} = 0,36 M, rt, 24 h					
Ligand ee (%)	Product ee (%) RUN 1	Product ee (%) RUN 2	Product ee (%) RUN 3	Final product ee (%)	Standard deviation
0	0	0	0	0.0	0.00
2.5	17.3	11.8	4.98	11.0	4.25
5	13.38	25.4	32	24.0	6.81
7.5	35.6	46.99	34.73	39.0	5.26
10	41.3	66.21	46.21	51.2	9.98
15	85.59	86.28	85.57	85.8	0.31
20	86.44	86.84	86.17	86.5	0.24
25	86.21	86.63	86.3	86.4	0.17
30	86.42	86.32	86.11	86.3	0.12
40	86.4	85.9	85.12	85.8	0.46
50	85.66	85.9	85.1	85.6	0.30
60	85.13	85.36	85.02	85.2	0.13
70	84.89	85.15	84.63	84.9	0.17
80	83.92	84.74	83.88	84.2	0.37
100	83.16	83.55	83.26	83.3	0.15
-20 °C					
At C_{PhCHO} = 0,36 M, rt, 24 h					
Ligand ee (%)	Product ee (%) RUN 1	Product ee (%) RUN 2	Product ee (%) RUN 3	Final product ee (%)	Standard deviation
0	0	0	0	0.0	0.00
2.5	16.5	20.5	6.8	15.0	5.20
5	17.92	27	45.51	30.1	10.24
7.5	51.7	49.94	55.2	52.3	1.95
10	58.3	67.41	71	65.6	4.85
15	87.4	87.43	87.69	87.5	0.12
20	88.5	87.78	89.23	88.5	0.48
25	88	87.59	88.97	88.2	0.52
30	88	87.02	88.54	87.9	0.56
40	87.8	86.3	88.31	87.5	0.78
50	87.1	86.12	88.64	87.3	0.90
60	87.2	86.1	88.19	87.2	0.71
70	86.4	86.1	87.74	86.7	0.66
80	85.8	86.19	87.97	86.7	0.88
100	84.95	83.35	84.78	84.4	0.67

Supplementary Table S8. Reported data from NLEs studies using $ZnMe_2$ at different temperatures.

ZnMe2					
20 °C					
At C_{PhCHO} = 0,36 M, rt, 72 h					
ligand ee (%)	Product ee (%) RUN 1	Product ee (%) RUN 2	Product ee (%) RUN 3	Final product ee (%)	Standard deviation
0	0	0	0	0	0.00
2.5	12.2	27.45	33.76	24	8.18
5	30.1	33.8	43.21	36	5.00
7.5	42.7	50	47.3	47	2.64
10	48.9	40.05	56.94	48.6	5.72
15	68.2	55.12	61.99	61.8	4.43
20	70	68.35	71.4	69.9	1.04
25	70.3	68.76	70.88	70.0	0.81
30	70.87	69.83	70.31	70.3	0.36
40	69	68.4	69.72	69.0	0.45
50	69	68.51	69.59	69.0	0.37
60	69.2	68.42	69.01	68.9	0.30
70	68	68.11	68.66	68.3	0.27
80	69	67.19	68.22	68.1	0.63
100	68.58	68.12	67.9	68.2	0.25
0 °C					
At C_{PhCHO} = 0,36 M, rt, 72 h					
ligand ee (%)	Product ee (%) RUN 1	Product ee (%) RUN 2	Product ee (%) RUN 3	Final product ee (%)	Standard deviation
0	0	0	0	0	0.00
2.5	9	17.9	12.3	13	3.22
5	12.95	20.1	26.19	20	4.53
7.5	30.12	33.18	36.72	33	2.25
10	37.87	28	45.1	37.0	5.99
15	63.49	66.3	66.98	65.6	1.40
20	72.74	71.28	74.85	73.0	1.26
25	76.07	74.15	79.3	76.5	1.86
30	72.24	76	80.57	76.3	2.87
40	73.38	75.68	78.77	75.9	1.88
50	71.4	70.8	72.73	71.6	0.72
60	72.54	69.2	73.25	71.7	1.64
70	68.58	68.1	69.03	68.6	0.31
80	68.4	66.9	67.8	67.7	0.53
100	66.34	66	66.64	66.3	0.22
-20 °C					
At C_{PhCHO} = 0,36 M, rt, 72 h					
ligand ee (%)	Product ee (%) RUN 1	Product ee (%) RUN 2	Product ee (%) RUN 3	Final product ee (%)	Standard deviation
0	0	0	0	0	0.00
2.5	18.6	8.4	14.62	14	3.65
5	25.34	22.9	33.85	27	4.32
7.5	41.55	45.29	39	42	2.23
10	59.51	52.2	61.2	57.6	3.62
15	60.76	61.76	62.78	61.8	0.68
20	62.2	63.8	64.61	63.5	0.89
25	64.68	64.6	65.23	64.8	0.26
30	64.23	65.1	65.7	65.0	0.52
40	59.76	61.4	64.21	61.8	1.61
50	56.01	60.5	62.84	59.8	2.52
60	58.31	60.1	62	60.1	1.24
70	58.69	59.55	56.2	58.1	1.30
80	56.07	57.74	55	56.3	0.98
100	54.06	54.32	54.9	54.4	0.32

b) Catalyst loading data

Supplementary Table S9. Reported data from catalyst loading screening using $ZnEt_2$ at different temperatures.

ZnEt2					
20 °C					
At C_{PhCHO} = 0,36 M, rt, 24 h					
Catalyst loading (mol%)	Product ee (%) RUN 1	Product ee (%) RUN 2	Product ee (%) RUN 3	Final product ee (%)	Standard deviation
2.5	87.12	87.39	87	87.2	0.15
5	86.5	86.5	84.8	85.9	0.76
10	84.19	83.74	83.37	83.8	0.28
15	82.5	82.43	82.11	82.3	0.16
20	81.15	81.78	81.35	81.4	0.24
0 °C					
At C_{PhCHO} = 0,36 M, rt, 24 h					
Catalyst loading (mol%)	Product ee (%) RUN 1	Product ee (%) RUN 2	Product ee (%) RUN 3	Final product ee (%)	Standard deviation
2.5	89.59	88.96	89.21	89.3	0.22
5	88.19	87.84	87.63	87.9	0.20
10	85.35	85.1	85.28	85.2	0.10
15	85.05	84.75	84.53	84.8	0.18
20	83.16	83.55	83.26	83.3	0.15
-20 °C					
At C_{PhCHO} = 0,36 M, rt, 24 h					
Catalyst loading (mol%)	Product ee (%) RUN 1	Product ee (%) RUN 2	Product ee (%) RUN 3	Final product ee (%)	Standard deviation
2.5	90.91	90	90.37	90.4	0.32
5	89.7	89.22	88.96	89.3	0.27
10	87.3	86.7	87.08	87.0	0.22
15	86.22	86.19	86.41	86.3	0.09
20	84.95	83.35	84.78	84.4	0.67

Supplementary Table S10. Reported data from catalyst loading screening using $ZnMe_2$ at different temperatures

ZnMe2					
20 °C					
At C_{PhCHO} = 0,36 M, rt, 72 h					
Catalyst loading (mol%)	Product ee (%) RUN 1	Product ee (%) RUN 2	Product ee (%) RUN 3	Final product ee (%)	Standard deviation
2.5	85.08	80.99	81.32	82.5	1.74
5	81.68	79.23	79.43	80.1	1.04
10	77	76.03	75.67	76.2	0.51
15	73.1	71.2	72.85	72.4	0.79
20	68.58	68.12	67.9	68.2	0.25
0 °C					
At C_{PhCHO} = 0,36 M, rt, 72 h					
Catalyst loading (mol%)	Product ee (%) RUN 1	Product ee (%) RUN 2	Product ee (%) RUN 3	Final product ee (%)	Standard deviation
2.5	78.73	77.98	77.15	78.0	0.54
5	75.93	77.5	77.86	77.1	0.78
10	72.36	72.7	73.23	72.8	0.31
15	69.03	68.6	66.8	68.1	0.90
20	66.34	66	66.64	66.3	0.22
-20 °C					
At C_{PhCHO} = 0,36 M, rt, 72 h					
Catalyst loading (mol%)	Product ee (%) RUN 1	Product ee (%) RUN 2	Product ee (%) RUN 3	Final product ee (%)	Standard deviation
2.5	71.85	70.12	70.16	70.7	0.76
5	66.81	66.3	68.02	67.0	0.65
10	61.75	60.2	60.99	61.0	0.52
15	56.15	57.36	57.12	56.9	0.48
20	54.06	54.32	54.9	54.4	0.32

c) Superimposition of ee_P as function of catalyst loading data (from Figure 7 in the paper)

Supplementary Table S11. Reported superimposition data from $ZnEt_2$ use

ZnEt2					
20 °C					
At C_{PhCHO} = 0,36 M, rt, 24 h					
Catalyst loading eq in eeL (%)	Product ee (%) RUN 1	Product ee (%) RUN 2	Product ee (%) RUN 3	Final product ee (%)	Standard deviation
12.5	87.12	87.39	87	87.2	0.15
25	86.5	86.5	84.8	85.9	0.76
50	84.19	83.74	83.37	83.8	0.28
75	82.5	82.43	82.11	82.3	0.16
100	81.15	81.78	81.35	81.4	0.24
20 °C					
At C_{PhCHO} = 0,36 M, rt, 24 h					
Ligand ee (%)	Product ee (%) Run 1	Product ee (%) Run 2	Product ee (%) Run 3	Final product ee (%)	Standard deviation
0	0	0	0	0.0	0.00
2.5	20.87	12.56	18.45	17.3	3.16
5	11.98	25.98	35.9	24.6	8.43
7.5	35.78	46.7	39.76	40.7	3.97
10	53	47.19	65.71	55.3	6.94
15	69.74	71	74.12	71.6	1.67
20	75.03	76.29	80.3	77.2	2.06
25	82.16	82.04	82.34	82.2	0.11
30	81.82	82.39	82.01	82.1	0.21
40	80.66	82.2	82	81.6	0.64
50	81.61	82.74	81.48	81.9	0.53
60	82.07	81.97	81.11	81.7	0.40
70	82.2	81.73	81.46	81.8	0.27
80	82.65	81.33	81.06	81.7	0.65
100	81.78	81.15	81.35	81.4	0.24
0 °C					
At C_{PhCHO} = 0,36 M, rt, 24 h					
Catalyst loading eq in eeL (%)	Product ee (%) RUN 1	Product ee (%) RUN 2	Product ee (%) RUN 3	Final product ee (%)	Standard deviation
12.5	89.59	88.96	89.21	89.3	0.22
25	88.19	87.84	87.63	87.9	0.20
50	85.35	85.1	85.28	85.2	0.10
75	85.05	84.75	84.53	84.8	0.18
100	83.16	83.55	83.26	83.3	0.15
0 °C					
At C_{PhCHO} = 0,36 M, rt, 24 h					
Ligand ee (%)	Product ee (%) RUN 1	Product ee (%) RUN 2	Product ee (%) RUN 3	Final product ee (%)	Standard deviation
0	0	0	0	0.0	0.00
2.5	17.3	11.8	4.98	11.0	4.25
5	13.38	25.4	32	24.0	6.81
7.5	35.6	46.99	34.73	39.0	5.26
10	41.3	66.21	46.21	51.2	9.98
15	85.59	86.28	85.57	85.8	0.31
20	86.44	86.84	86.17	86.5	0.24
25	86.21	86.63	86.3	86.4	0.17
30	86.42	86.32	86.11	86.3	0.12
40	86.4	85.9	85.12	85.8	0.46
50	85.66	85.9	85.1	85.6	0.30
60	85.13	85.36	85.02	85.2	0.13
70	84.89	85.15	84.63	84.9	0.17
80	83.92	84.74	83.88	84.2	0.37
100	83.16	83.55	83.26	83.3	0.15
-20 °C					
At C_{PhCHO} = 0,36 M, rt, 24 h					
Catalyst loading eq in eeL (%)	Product ee (%) RUN 1	Product ee (%) RUN 2	Product ee (%) RUN 3	Final product ee (%)	Standard deviation
12.5	90.9	90	90.37	90.4	0.32
25	89.7	89.22	88.96	89.3	0.27
50	87.3	86.7	87.08	87.0	0.22
75	86.2	86.19	86.41	86.3	0.10
100	84.9	83.35	84.78	84.3	0.66
-20 °C					
At C_{PhCHO} = 0,36 M, rt, 24 h					
Ligand ee (%)	Product ee (%) RUN 1	Product ee (%) RUN 2	Product ee (%) RUN 3	Final product ee (%)	Standard deviation
0	0	0	0	0.0	0.00
2.5	16.5	20.5	6.8	15.0	5.20
5	17.92	27	45.51	30.1	10.24
7.5	51.7	49.94	55.2	52.3	1.95
10	58.3	67.41	71	65.6	4.85
15	87.4	87.43	87.69	87.5	0.12
20	88.5	87.78	89.23	88.5	0.48
25	88	87.59	88.97	88.2	0.52
30	88	87.02	88.54	87.9	0.56
40	87.8	86.3	88.31	87.5	0.78
50	87.1	86.12	88.64	87.3	0.90
60	87.2	86.1	88.19	87.2	0.71
70	86.4	86.1	87.74	86.7	0.66
80	85.8	86.19	87.97	86.7	0.88
100	84.9	83.35	84.78	84.3	0.66

Supplementary Table S12. Reported superimposition data from $ZnMe_2$ use

ZnMe2					
20 °C					
At C_{PhCHO} = 0,36 M, rt, 72 h					
Catalyst loading eq in eeL (%)	Product ee (%) RUN 1	Product ee (%) RUN 2	Product ee (%) RUN 3	Final product ee (%)	Standard deviation
12.5	85.08	80.99	81.32	82.5	1.74
25	81.68	79.23	79.43	80.1	1.04
50	77	76.03	75.67	76.2	0.51
75	73.1	71.2	72.85	72.4	0.79
100	68.58	68.12	67.9	68.2	0.25
20 °C					
At C_{PhCHO} = 0,36 M, rt, 72 h					
Ligand ee (%)	Product ee (%) Run 1	Product ee (%) Run 2	Product ee (%) Run 3	Final product ee (%)	Standard deviation
0	0	0	0	0.0	0.00
2.5	12.2	27.45	33.76	24.5	8.18
5	30.1	33.8	43.21	35.7	5.00
7.5	42.7	50	47.3	46.7	2.64
10	48.9	40.05	56.94	48.6	5.72
15	68.2	55.12	61.99	61.8	4.43
20	70	68.35	71.4	69.9	1.04
25	70.3	68.76	70.88	70.0	0.81
30	70.87	69.83	70.31	70.3	0.36
40	69	68.4	69.72	69.0	0.45
50	69	68.51	69.59	69.0	0.37
60	69.2	68.42	69.01	68.9	0.30
70	68	68.11	68.66	68.3	0.27
80	69	67.19	68.22	68.1	0.63
100	68.58	68.2	67.9	68.2	0.24
0 °C					
At C_{PhCHO} = 0,36 M, rt, 72 h					
Catalyst loading eq in eeL (%)	Product ee (%) RUN 1	Product ee (%) RUN 2	Product ee (%) RUN 3	Final product ee (%)	Standard deviation
12.5	78.73	77.98	77.15	78.0	0.54
25	75.93	77.5	77.86	77.1	0.78
50	72.36	72.7	73.23	72.8	0.31
75	69.03	68.6	66.8	68.1	0.90
100	66.34	66	66.64	66.3	0.22
0 °C					
At C_{PhCHO} = 0,36 M, rt, 72 h					
Ligand ee (%)	Product ee (%) RUN 1	Product ee (%) RUN 2	Product ee (%) RUN 3	Final product ee (%)	Standard deviation
0	0	0	0	0.0	0.00
2.5	9	17.9	12.3	13.0	3.22
5	12.95	20.1	26.19	20.0	4.53
7.5	30.12	33.18	36.72	33.0	2.25
10	37.87	28	45.1	37.0	5.99
15	63.49	66.3	66.98	65.6	1.40
20	72.74	71.28	74.85	73.0	1.26
25	76.07	74.15	79.3	76.5	1.86
30	72.24	76	80.57	76.3	2.87
40	73.38	75.68	78.77	75.9	1.88
50	71.4	70.8	72.73	71.6	0.72
60	72.54	69.2	73.25	71.7	1.64
70	68.58	68.1	69.03	68.6	0.31
80	68.4	66.9	67.8	67.7	0.53
100	66.34	66	66.64	66.3	0.22
-20 °C					
At C_{PhCHO} = 0,36 M, rt, 72 h					
Catalyst loading eq in eeL (%)	Product ee (%) RUN 1	Product ee (%) RUN 2	Product ee (%) RUN 3	Final product ee (%)	Standard deviation
12.5	71.85	70.12	70.16	70.7	0.76
25	66.81	66.3	68.02	67.0	0.65
50	61.75	60.2	60.99	61.0	0.52
75	56.15	57.36	57.12	56.9	0.48
100	54.06	54.32	54.9	54.4	0.32
-20 °C					
At C_{PhCHO} = 0,36 M, rt, 72 h					
Ligand ee (%)	Product ee (%) RUN 1	Product ee (%) RUN 2	Product ee (%) RUN 3	Final product ee (%)	Standard deviation
0	0	0	0	0.0	0.00
2.5	18.6	8.4	14.62	14.0	3.65
5	25.34	22.9	33.85	27.4	4.32
7.5	41.55	45.29	39	41.9	2.23
10	59.51	52.2	61.2	57.6	3.62
15	60.76	61.76	62.78	61.8	0.68
20	62.2	63.8	64.61	63.5	0.89
25	64.68	64.6	65.23	64.8	0.26
30	64.23	65.1	65.7	65.0	0.52
40	59.76	61.4	64.21	61.8	1.61
50	56.01	60.5	62.84	59.8	2.52
60	58.31	60.1	62	60.1	1.24
70	58.69	59.55	56.2	58.1	1.30
80	56.07	57.74	55	56.3	0.98
100	54.06	54.32	54.9	54.4	0.32

d) Temperature data

Supplementary Table S13. Reported data from the temperature screening

ZnEt2						
At C_{PhCHO} = 0,36 M, 20mol%, 72 h						
Temperature (°C)	Product ee (%) RUN 1	Product ee (%) RUN 2	Product ee (%) RUN 3	Final ee (%)	Temperature deviation (°C)	Standard deviation
-20	84.95	83.35	84.78	84.4	2	0.67
-10	83.90	84.01	84.10	84.0	2	0.07
0	83.16	83.55	83.26	83.3	2	0.15
20	81.15	81.78	81.35	81.4	5	0.24
40	77.90	77.77	77.59	77.8	2	0.11
ZnMe2						
At C_{PhCHO} = 0,36 M, 20mol%, 72 h						
Temperature (°C)	Product ee (%) RUN 1	Product ee (%) RUN 2	Product ee (%) RUN 3	Final ee (%)	Temperature deviation	Standard deviation
-20	54.06	54.32	54.9	54.4	2	0.32
-10	60.28	56.54	59.44	58.8	2	1.48
0	66.34	66	66.64	66.3	2	0.22
20	68.58	68.12	67.9	68.2	5	0.25
40	75.1	72.12	69.91	72.4	2	1.82
